# Universal selenium nanoadjuvant with immunopotentiating and redox-shaping activities inducing high-quality immunity for SARS-CoV-2 vaccine

**DOI:** 10.1038/s41392-023-01371-1

**Published:** 2023-02-27

**Authors:** Haoqiang Lai, Ligeng Xu, Chang Liu, Sujiang Shi, Yalin Jiang, Yangyang Yu, Bo Deng, Tianfeng Chen

**Affiliations:** Department of Oncology, The First Affiliated Hospital, Department of Chemistry, Jinan University, Guangzhou, 510632 China

**Keywords:** Drug delivery, Infectious diseases

**Dear Editor**,

The COVID-19 pandemic still greatly threatens the public health worldwide and novel vaccines to highly effectively combat SARS-CoV-2 remains an unmet clinical need. However, redox imbalance in immune cells under extra stimulation by vaccine or pathogens greatly dampens the quality of the induced immunity.^1^ In addition, people infected with COVID-19 that characterized by immune system malfunction were also approved to be attributed by the robust oxidative stress,^2^ indicating the crucial roles of regulation of redox balance in the metabolic rewiring of immune cells and its activation. Therefore, the development of novel vaccines with redox regulation and superior immuno-potentiating properties to combat against SARS-CoV-2 will be greatly desired.

Selenium (Se) as the essential trace element for humans possesses excellent antioxidant properties and exhibits critical roles in modulating activities of immune cells such as T_FH_ cells to promote antibody responses.^3,4^ However, the chemical form of selenium greatly determines its biological functions. Herein, different Se species including selenocysteine (SeCys2), selenomethionine (SeMet), selenite (Na_2_SeO_3_), EbSelen, and selenium nanoparticles (SeNPs) (Supplementary Fig. S1a) were selected for investigating their potential roles in regulating dendritic cells (DCs). We found that different Se species exhibited diverse modulating effects on DCs maturation, OT-I/II T cells proliferation and Th1 type cytokines (IFN-γ and TNF-α) secretion while SeNPs and SeMet possessing better safety on BMDCs ((Fig. 1a, Supplementary Fig. S1a–h). Intriguingly, SeNPs pretreatment was more efficiently to suppress LPS-induced intracellular ROS production (Fig. 1b, Supplementary Fig. S1i) with the involvement of upregulating antioxidant selenoproteins expression such as GPX1-4 and TrxR1-2 (Fig. 1c, Supplementary Fig. S2). These results suggest the regulation roles of SeNPs in shaping the redox state in DCs upon pathogens stimulation. Consequently, SeNPs were selected as adjuvant to construct RBD antigen-loaded nanovaccine (RBD@SeNPs) through electrostatic interaction with optimized formulation of RBD: Se weight ratio being 1:2. RBD@SeNPs showed average size at 100 nm and good stability (Supplementary Fig. S3). Intriguingly, Se nanoadjuvant enhanced the immunogenicity of both RBD and S1 spike protein antigens via activating DCs (Fig. 1d, Supplementary Fig. S4a–d) and promoted the generation of T_FH_ lymphocytes and germinal center B (GC B, FAS^+^GL7^+^) cells, which suggests the immunity potentiated capacity of Se on antigens to trigger robust antibody response (Supplementary Fig. S5). Further studies revealed that Se neoadjuvant was able to upregulate TLR1/2/4 expressions, related adaptor proteins (Myd88, TRAM and TRIF) and its downstream molecules (Fig. 1e, f and Supplementary Fig. S4e), indicating the underlying molecular mechanisms of Se nanoadjuvant in promoting the immunogenicity of antigen was related to TLRs-mediated signaling pathways activation.

**Fig. 1:**
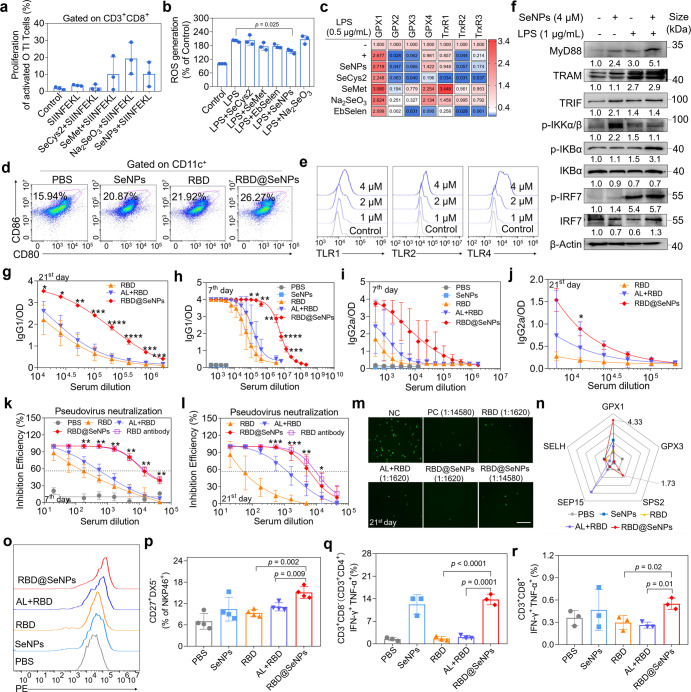
Se nanoadjuvant-based vaccine can highly effectively induce memory NK cells, Th1-biased immunity, and superior antigen-specific neutralizing antibodies with high titer. **a** The effects of Se species (4 μM) on the functions of bone marrow-derived dendritic cells (BMDCs) pulsed with SIINFEKL (1.5 μg/mL) to induce the proliferation of T cells from OT-I mice. Briefly, BMDCs were co-treated with SIINFEKL peptides and different Se species for 12 h and then incubated with CFSE-labeled splenocytes from OT-I mice for 3 days. After that, the CD8^+^ T cells proliferation efficacy was analyzed using flow cytometry assay. **b** Effects of Se species on ROS generation within BMDCs under LPS stimulation. BMDCs (1 × 10^6^ cells/mL) were pre-treated with Se species (4 μM) overnight and then stained with DCFH-DA probe (10 μM). After rinsed with PBS twice, LPS was added into cells and the fluorescence was recorded. **c** Effects of Se species on mRNA expressions of selenoproteins in BMDCs under the stimulation of LPS using qPCR analysis. BMDCs cells were pretreated with different Se species (4 μM) for 2 h and then co-incubated with LPS overnight. After that, mRNA expressions of selenoproteins were analyzed using qPCR assay. **d** Effects of RBD@SeNPs and other different treatments on the maturation of DCs in inguinal lymph nodes at day 3 post vaccination. **e** Effects of Se nanoadjuvant at different concentrations on the expression of TLRs (TLR1, 2, 4) on the surface of BMDCs using flow cytometry analysis. **f** Effects of Se nanoadjuvant on the expressions of TLRs downstream signaling pathways proteins using western blotting analysis. The antibody titer of IgG2a (**g**, **i**) and IgG1 (**h**, **j**) against RBD antigen at day 7 and 21 post final immunization using ELISA assay, respectively. The inhibition efficacy of neutralizing antibody in serum collected at day 7 (**k**) and day 21 (**l**) post final immunization against SARS-CoV-2 spike pseudovirus. Sera of series dilution were co-incubated with SARS-CoV-2 spike pseudovirus for 1 h. After that, the treated pseudovirus was incubated with 293T-ACE2 target cells for 48 h at 37 °C. The dash lines indicated the half maximal inhibitory efficiency against SARS-CoV-2 Spike pseudovirus. **m** Representative images of 293T ACE2 cells incubated with pseudovirus which treated with serum collected at day 21 post final immunization from different groups. Scale bar, 200 μm. **n** The mRNA expression of GPX1, GPX3, SPS2, SEP15 and SELH in splenocytes of different groups at day 7 post final immunization. **o** Effects of different vaccinations on the phagocytosis capability of macrophages from immunized mice. Peritoneal macrophages were collected at day 7 after the final vaccination and incubated with fluorescence latex beads (100 nm, 2.5 μg/mL) overnight. The phagocytosis capabilities were determined by quantifying the intracellular fluorescence intensity using flow cytometry assay. **p** Effects of different vaccinations on the generation of memory NK cells. Co-expression of IFN-γ and TNF-α in CD3^+^CD8^-^ (CD3^+^CD4^+^) (**q)** and CD3^+^CD8^+^ T cells (**r**) in splenocytes from immunized mice. Splenocytes from immunized mice were collected at day 7 post final immunization and stimulated with 5 μg/mL recombinant RBD antigen and protein secretion inhibitors (Brefeldin A and Monensin) for 6 h. Then, splenocytes were stained with specific antibodies against cytokines. Data are expressed as the mean ± SD. *n* = 3 per group in **a**–**e** and **q**–**r**. *n* = 4 in **g**–**p**. *p < 0.05, **p < 0.01, ***p < 0.001, ****p < 0.0001 by one-way ANOVA

To evaluate the immunogenicity of Se nanoadjuvant-based vaccine, mice were immunized with RBD@SeNPs through intradermal injection (Supplementary Fig. S6a). It was found that Se-based nanovaccine exhibited the highest activities in inducing IgM, IgG1, and IgG2a antibody with higher titers compared to RBD and AL + RBD immunization groups at day 7 post final immunization (Fig. 1g, h and Supplementary Fig. S6b). The IgG2a and IgG1 antibody titers in serum of RBD@SeNPs group were 16 and 32 times than that of AL + RBD immunization modality, respectively. Actually, SeNPs immunization did not induce any generations of RBD-specific antibody, similar to PBS group. In other words, SeNPs as adjuvant could effectively promote the humoral immunity induced by RBD antigen. As one of the typical hallmarks of vaccines, antigen-specific antibody with high titer in long term plays important roles in combating against pathogens invasion. Since IgM produced immediately upon exposure to pathogens, it will dramatically decline and replaced by IgG after long time. Intriguingly, only the IgM antibody of RBD@SeNPs immunization group was still in relatively high titer while that of both RBD and AL + RBD treatment groups dramatically declined at day 21 post final immunization (Supplementary Fig. S6c). Furthermore, RBD@SeNPs immunization group could endow both IgG2a and IgG1 with higher titer than that of RBD and AL + RBD groups (Fig. 1i and j). In addition, Th1/Th2 balance analysis demonstrated that the ratio of IgG2a to IgG1 of RBD@SeNPs immunization was much higher than that of both RBD and AL + RBD treatments (Supplementary Fig. S6d), indicating the unique advantage of the Se-based nanovaccine in inducing both cellular (Th1-biased) and humoral immune responses. All those unique properties of the developed vaccine will be greatly useful for antibody-dependent cellular cytotoxicity (ADCC) to effectively combat and eliminate the SARS-CoV-2 infection.

In order to explore the potential protection effects induced by Se-based nanovaccine immunization against virus infection, SARS-CoV-2 pseudo-virus neutralization assay was employed. Excitingly, the serum of mice immunized with Se-based nanovaccine exhibited comparable inhibitory activity to the commercial RBD antibody even at day 21 post final vaccination, which was about 27 folds higher than that of AL + RBD immunization (Fig. 1k, l). These results were also confirmed by the fluorescence images obtained from 293 T ACE2 cells in pseudo-virus neutralization assay (Fig. 1m, Supplementary Fig. S7). Evidence has disclosed the critical roles of selenoprotein in potentiating antibody response through restraining oxidative stress.^3^ We found that RBD@SeNPs immunization could significantly induce the up-regulation of GPX1 and SPS2 (Fig. 1n, Supplementary Fig. S8). These results may suggest that Se nanoadjuvant can effectively deliver the antigen and regulate the expressions of selenoproteins with redox modulation activities to satisfy the demands of stimulated immune cells for rapid proliferation, robust cytokines generations and shape the immune system to combat pathogens infection.

The cross talking between innate immunity and adaptive immune responses is absolutely desired to highly effectively combat acute pathogens infection. It was found that RBD@SeNPs vaccination could significantly enhance the phagocytosis capability of peritoneal macrophages and stimulate the generation of memory NK cells at day 7 post final immunization (Fig. 1o, p and Supplementary Fig. S9a, b). In addition, the nanovaccine enhanced the proliferation capabilities of both CD4^+^ and CD8^+^ T lymphocytes (Supplementary Fig. S9c, d). As the typical markers of Th1-biased immune responses, TNF-α and IFN-γ play important roles in regulating innate and adaptive immunities to combat pathogen infection. RBD@SeNPs vaccination was found to exhibit higher activities in promoting the multi-functionality of CD3^+^CD8^-^ (CD3^+^CD4^+^) and CD3^+^CD8^+^ T lymphocytes to simultaneously produce both TNF-α and IFN-γ than RBD and AL + RBD treatment groups (Fig. 1q, r and Supplementary Fig. S9e–h). All those solid evidences indicate that Se nanoadjuvant-based vaccine has its own unique advantages in triggering Th1-biased immune responses. The immunological memory is the hallmark feature of vaccines to prevent humans from the second infection.^5^ Actually, the nanovaccine did not induce more generation of memory T/B cells compared to RBD and AL + RBD immunization groups (Supplementary Fig. S10) and further studies to optimize the design is still required, which is always the big challenge for nonviral-based vaccines. As expected, the histopathological analysis of major organs involving heart, liver, spleen, lung and kidney (Supplementary Fig. S11) and no significant changes of both IL-4 and IL-21 expressions (the typical markers of immune-related adverse events, irAEs) indicated good safety profiles of Se nanoadjuvant-based vaccines (Supplementary Fig. S12).

In summary, Selenium nanoadjuvant which exhibiting both immuno-potentiating and redox-shaping activities to regulate GPXs selenoproteins expression within immune cells can efficiently improve its functionality and responsiveness to vaccination. Besides activating innate immune cells, Se nanoadjuvant-based vaccine can also induce Th1-biased immunity and superior antigen-specific neutralizing antibodies with high titer and high efficacy to combat pseudovirus infection (Supplementary Fig. S13). All those evidences make selenium nanoadjuvant as a universal candidate agent to facilitate immune system against major diseases.

## Supplementary information

Supplemental material-Clean version

## Data Availability

All data needed to evaluate the conclusions in the paper are present in the paper or the Supplementary Materials. Materials described in the study are either commercially available or on request from the corresponding author.
