# Supplementary material for: Universal selenium nanoadjuvant with immunopotentiating and redox-shaping activities inducing high-quality immunity for SARS-CoV-2 vaccine
Source: Signal Transduct Target Ther. 2023 Feb 27;8:88. doi: 10.1038/s41392-023-01371-1 (PMC9969362; doi:10.1038/s41392-023-01371-1)
Supplement: Supplementary file 1 — Supplemental material-Clean version [file 41392_2023_1371_MOESM1_ESM.docx]

Supplementary Materials for

**Universal selenium nanoadjuvant with immunopotentiating and redox-shaping activities inducing high-quality immunity for SARS-CoV-2 vaccine**

Haoqiang Lai, Ligeng Xu,* Chang Liu, Sujiang Shi, Yalin Jiang, Yangyang Yu, Bo Deng, Tianfeng Chen*

*Corresponding author: lgxu19@jnu.edu.cn, tchentf@jnu.edu.cn

**This PDF file includes:**

Materials and Methods

The abbreviation table

Figs. S1 to S13

Table S1

**Materials and Methods**

**Materials**

The recombinant RBD protein was obtained from Sino Biological (Cat: 40592-V08H). Ascorbic acid and sodium selenite were purchased from Sigma Aldrich. Lentinan was obtained from Topscience Co. Ltd. Antibodies such as FITC-CD3, APC-Cy7-CD4, APC-CD8, PE-CD44, e450-CD62L, Pacific blue-CD19, FITC-CD27, APC-CD40, FITC-Nkp46, PerCP-Cy5.5-DX5, PE-Cy7-CD27, BV421-CXCR6, APC-IFN-γ and APC-A750-TNF-α were purchased from Biolegend. HRP conjugated IgM, IgG1 and IgG2a were obtained from Bethyl laboratories. Inc. DCFH-DA probe was obtained from Sigma. The primers of GPX, TRXR, SELH and SPS2 were obtained from Sangon Biotech Co., Ltd. (Shanghai, China).

**RBD@SeNPs vaccine synthesis and characterizations**

Sodium selenite solution (10 mM) in autoclaved water was mixed with lentinan (20 mg/mL) for 30 min. After that, ascorbic acid solution (40 mM) was added by dropwise and the mixture were stirring at the condition of 4 ℃ overnight. Finally, the sample was obtained after dialyzing against autoclaved water using a 20 kDa MWCO membrane and then the synthesized SeNPs was subjected for the atomic fluorescence spectrometer assay to obtain the content of Se. BCA (Beyotime Biotechnology (P0010S)) assay was employed to figure out the best coupling efficiency of RBD and SeNPs. Briefly, RBD and SeNPs were mixed at different weight ratio of 1: 1, 1: 2 and 1: 4 (μg/μg) under room temperature for 30 min. After the reaction, the products were centrifuged at 12000 g for 30 min and the supernatant were subjected for BCA assay to quantify the concentration of protein and then the coupling efficiency was calculated as E (efficacy) = (total RBD – residual RBD)/total RBD × 100%. The purified SeNPs and RBD recombinant protein (or S1 recombinant protein) were mixed at the ratio of 1: 2 (w/w) and stirring at the condition of 4 ℃ for 3 h to obtain RBD@SeNPs. The final products were characterized using transmission electron microscope, high resolution transmission electron microscope and atomic force microscope.

**Evaluation of Dendritic cells (DCs) maturation**

Bone marrow derived dendritic cells (BMDCs) were isolated from 8-week-old C57BL/6 mice and cultured in RPMI-1640 medium (Gibco, Thermo Fisher Scientific, MA, USA) supplemented with 10% fetal bovine serum, 50 μM 2-mercaptoethanol (Sigma) and 20 ng/mL GM-CSF (Peprotech/Tebu, Frankfurt, Germany). Then, cells (5 × 10^5^ cells/well) were treated with 4 μM SeCys2, SeMet, SeNPs, Na_2_SeO_3_ and EbSelen for 24 h, respectively. After that, cells were collected and then stained with FITC-CD11c, PE-CD80 and APC-A750-CD86 antibodies for 30 min at room temperature, and then cells were subjected for flow cytometry assay to quantify the population of the mature BMDCs. For *in vivo* evaluation of DCs maturation, mice were vaccinated with RBD (20 μg), S1 (20 μg), SeNPs (40 μg), RBD@SeNPs (RBD, 20 μg and Se, 40 μg) and S1@SeNPs (S1, 20 μg and Se, 40 μg). After the immunization for 2-3 days, cells in inguinal lymph nodes were obtained and stained with Bv421-CD45, APC-CD11c, PE-CD80 and APC-A750-CD86 for 30 min. CD80^+^CD86^+^ DCs were gated from CD11c positive cells derived from CD45^+^ cells.

**Proliferation evaluation of** **OT I and OT II T cells**

Splenocytes from OT I and OT II mice (purchased from Cyagen Biosciences Inc. and breeding at Laboratory Animal center of Jinan University. OT I and OT II mice grow in individually ventilated cages at 16-20 ℃, 40-70% humidity condition and received with 12 hours of light per day). Splenocytes were collected from OT-I and OT-II mice and stained separately with carboxyfluorescein succinimidyl ester (CFSE) (Invitrogen) as per the manufactory protocol. Briefly, cells were incubated with 10 μM CFSE for 15 min at 37 °C. After three washes with RPMI-1640 medium, 1 × 10^6^ cells were incubated with or without SIINFEKL (1.5 μg/mL) and OVA_323-339_ (5 μg/mL) in combined with BMDC that pretreated with 4 μM SeCys2, SeMet, SeNPs, Na_2_SeO_3_ and EbSelen for 24 h, respectively. After 3 days incubation, splenocytes were collected and stained with PE-Cy7 CD3, PC-CD8, APC-Cy7-CD4 antibody (Biolegend) for 30 min at room temperature. After washed with PBS twice, cells were analyzed by flow cytometry (Cytoflex, Beckman) assay and the decreased fluorescent intensity of CFSE-labeled cells in the population of CD3^+^CD8^+^ T cells or CD3^+^CD4^+^ T were quantified as the proliferated cells.

**Vaccination**

The RBD protein was mixed with Imject Alum at 1: 1 of volume ratio and incubated at 4 ℃ for 1 h according to the manufactory instructions. BALB/c mice were injected intradermally with recombinant aluminum adjuvant-assistant RBD vaccine (20 μg RBD protein per mouse), RBD protein (20 μg per mouse), SeNPs (40 μg Se per mouse), RBD@SeNPs (20 μg RBD protein and 40 μg Se per mouse) and PBS at two weeks interval. Sera from immunized mice were collected at day 7 and 21 post final immunization and applied for IgM, IgG1, IgG2a titration and SARS-Cov-2 pseudovirus neutralization analysis. Additionally, splenocytes from the immunized mice were collected for evaluating the intracellular cytokine expression, such as IFN-γ and TNF-α, and the population of memory T cells, memory B cells and memory NK cells by specific antibody staining using flow cytometry assay.

**Neutralization of** **SARS-CoV-2 Spike pseudovirus**

The SARS-CoV-2 Spike Pseudovirus neutralization analysis were carried by OBiO Technology (Shanghai) Corp.,Ltd. Neutralization of SARS-CoV-2 pseudovirus assay was carried out by measuring the expression of luciferase encoded by pLenti-CMV-EGFP-3xFLAG-EF1-Luc-WPRE pseudovirus (OBio scientific service) after transfected into ACE2-transfected HEK293T cells (293T-ACE2 cells). Briefly, different fold dilutions of serum of immunized mice from different treatment groups were mixed with pseudovirus at 37 ℃ for 1 h. Then, 293T-ACE2 cells were added into the well containing pseudovirus mixture and incubated at 37 ℃ and 5% CO_2_ atmosphere for 48 h, the infected cells were taken photos by fluorescent microscopy (DMi8，LEICA) and the supernatant was aspirated and discarded from the culture plate. Then, cells were treated with cell lysis buffer for 5 min and the cell lysate was transferred to another new 96-well and incubated with luciferase substrate. Finally, the fluorescence intensity was recorded by fluorescence microplate reader (DMi8, LEICA)). After 48 hours, the infected cells were examined by fluorescent microscopy and then aspirated and discarded the supernatant from the culture plate, and lysed cells with cell lysis buffer for 5 min. Transferred the cell lysate to another new 96-well and added luciferase substrate to each well. The fluorescence intensity was recorded by fluorescence microplate reader, and the inhibition ratio was calculated as the following formular: Inhibition ratio = [1 - (Value _sample_ - Value _blank_) / (Value _negative_ - Value _blank_)] * 100%.

**RBD-specific antibody titer analysis**

Recombinant RBD protein was coated on a 96-well plate (Costar, Corning, Inc.USA) at a final concentration of 5 μg/mL in PBS at 4 ℃ overnight. After three washes with PBST (PBS containing 0.05% Tween-20), the plate was blocked with 1% BSA in PBST at 37 ℃ for 2 h. The plate was washed with PBST three times and incubated with serially diluted serum, such as 400-, 800-, 1600-, 3200, 6400, 12800-, 25600-, 51200-, 102400-, 204800-, 409600-, 819200-, 1638400-fold dilutions, at 37 ℃ for 2 h, and then the plate was rinsed with PBST three times. Antibodies, including HRP-linked goat anti-mouse IgM, IgG1 and IgG2a was added at 1:10000. After incubation for 1 h at 37 ℃, the plate was washed with PBST 4 times and added with TMB substrate solution for 10 min. Finally, the reaction was stopped with 1 N H_2_SO_4_ solution and the absorbance was recorded by a microplate reader at 450 nm.

**Flow cytometry assay**

Intracellular cytokines staining was performed by multicolor cytometry assay. Briefly, vaccinated mouse-derived splenocytes were stimulated with recombinant RBD (5 μg/mL) at the presence of brefeldin A and ionomycin for 6 hours at 37 °C and then cells were stained with FITC-CD3 and PE-CD8 antibody for surface staining for 30 min at room temperature. Cells were then permeabilized and labeled with anti-IFN-γ and TNF-α for another 30 min. Finally, cells were subjected for analyzing the expression of IFN-γ and TNF-α or co-expression in cells by employing Beckman flow cytometry assay (Beckman, Cytoflex), and at least 200,000 lymphocytes were collected for the analysis. For the evaluation of the population of memory T, memory B and memory NK cells, splenocytes were stained with specific antibody, respectively. Splenocytes were collected at day 21 after the final vaccination. After RBC lysis, cells were collected and subjected for immune staining. Briefly, 1 × 10^6^ cells were incubated with antibodies for 30 min at room temperature. The central memory and effector memory T cells are defined as CD44^+^CD62L^+^ and CD44^+^CD62L^-^ cells gated from CD3^+^ CD4^+^/CD8^+^ cells, respectively. The memory B cells were denoted as CD27^+^ and CD40^+^ double positive cells in CD3^-^CD19^+^ B cells. Meanwhile, NKP46^+^and DX5^-^ cells grated from CD3^-^ NK1.1^+^ were classified as memory NK cells. CXCR5^+^ ICOS^+^ (T_FH_) cells were gated from CD3^+^CD4^+^ T cells after the exclusion of B cells. GC B (FAS^+^GL7^+^) cells were derived from B cells that stained with APC-GL7 and PE-FAS.

**Quantitative Real-time PCR**

BMDCs (1×10^6^ cells/mL) were treated with different Se species (4 μM) for 2 h, prior to LPS (0.5 μg/mL) for 12 h at 37 ℃ atmosphere. Then cells were collected and the total RNA was extracted by Trizol. After reverse transcription (Accurate Biotechnology (Hunan) Co. Ltd), SYBR Green qPCR SuperMix-UDG (Invitrogen) was used for real-time PCR analysis as per the manufactory description. For the analysis of selenoprotein expression after vaccination, splenocytes from mice that received with the last dose of RBD@SeNPs at day 7 were collected and total RNA was extracted and used for Q-PCR anaylsis. The primer sequences of selenoproteins are available at Table S1.

**Statistical analysis**

Data are expressed as the mean ± standard deviation (SD). One Way ANOVA in SPSS Statistics 25 (SPSS Statistics 25; SPSS, Inc., Chicago, IL, USA) was applied for statistical analysis. **P* < 0.05 and ***P* < 0.01 were represented as significant differences among different groups.

**Supplementary Figures**

**The abbreviation table**

| **Abbreviation** | **Full name** |
| --- | --- |
| BMDC | Bone marrow-derived dendritic cells |
| CFSE | Carboxifluorescein diacetate succinimidyl ester |
| ROS | Reactive oxygen species |
| LPS | Lipopolysaccharide |
| DCFH-DA | 2’-7’dichlorofluorescin diacetate |
| TLR | Toll-like receptor |
| GPX | Glutathione peroxidase |
| SPS2 | Selenophosphate synthetase 2 |
| SEP15 | 15 kDa selenoprotein |
| SELH | Selenoprotein H |
| NK | Natural killer cells |
| RBD | Receptor-binding domain |
| S1 | Spike protein 1 |
| AL+RBD | Aluminum adjuvant mixed with RBD antigen |
| SeNPs | Selenium nanoparticles |
| SeMet | Selenomethionine |
| NC | Negative control |
| PC | Positive control (i.e., commercial anti-RBD antigen antibody) |


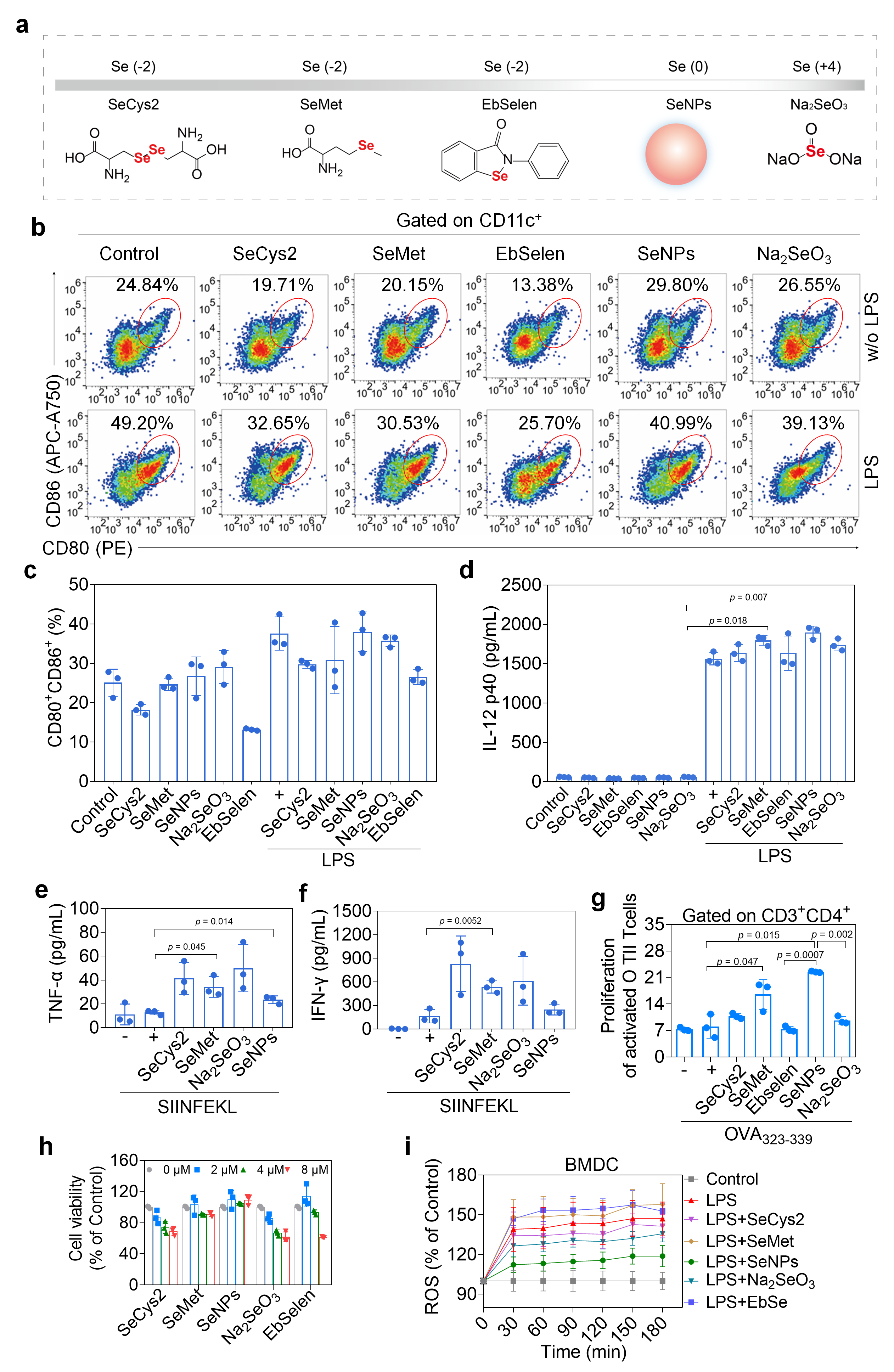


Fig. S1 Se species as chaperone facilitate the responsiveness and functions of dendritic cells to exogenous stimuli by shaping the cellular redox balance. a Different chemical forms of Se species. b Selenium (4 μM) regulates the maturation of dendritic cells under the stimulation of LPS (0.5 μg/mL). BMDCs were pretreated with 4 μM selenium for 2 h and then co-treated with LPS for 12 h. c Effects of Se species on the maturation of BMDCs and its responsiveness to LPS stimulation. Data are represented as mean ± SD, n = 3 per group. d The expression level of IL-12p40 of dendritic cells after treatment with different Se species (4 μM) and LPS (0.5 μg/mL). Effects of BMDC treated with Se species (4 μM) and SIINFEKL (1.5 μg/mL) on the expression of TNF-α (e) and IFN-γ (f). BMDCs were pretreated with selenium for 12 h and then added into CFSE-labeled OT I T cells alone or in combined with SIINFEKL for 3 days. After that, cells were applied for proliferation assay and the culture medium was used for evaluating the expression level of IFN-γ and TNF-α. g Effects of BMDC treated with Se species (4 μM) and OVA_323-339_ (5 μg/mL) on the proliferation of OT-II T cell. BMDCs were pretreated with selenium for 12 h and then added into CFSE-labeled OT II T cells alone or in combined with OVA_323-339_ for 3 days. After that, cells were applied for proliferation assay. n=3 per group. h Cytotoxicity effects of different Se species on BMDCs. BMDCs were treated with different concentration of Se species for 24 h and the cell viability was examined by CCK8 assay. Data are expressed as the mean ± SD, n = 3 per group. i Effects of selenium on ROS generation within BMDCs. Changes of DCF fluorescence were recorded for 3 h. Data are expressed as the mean ± SD. n = 3 per group.

**Fig.S2** Effects of selenium species on mRNA expressions of selenoproteins in BMDCs under LPS stimulation using qPCR analysis. Data are expressed as the mean ± SD. n = 3 per group.


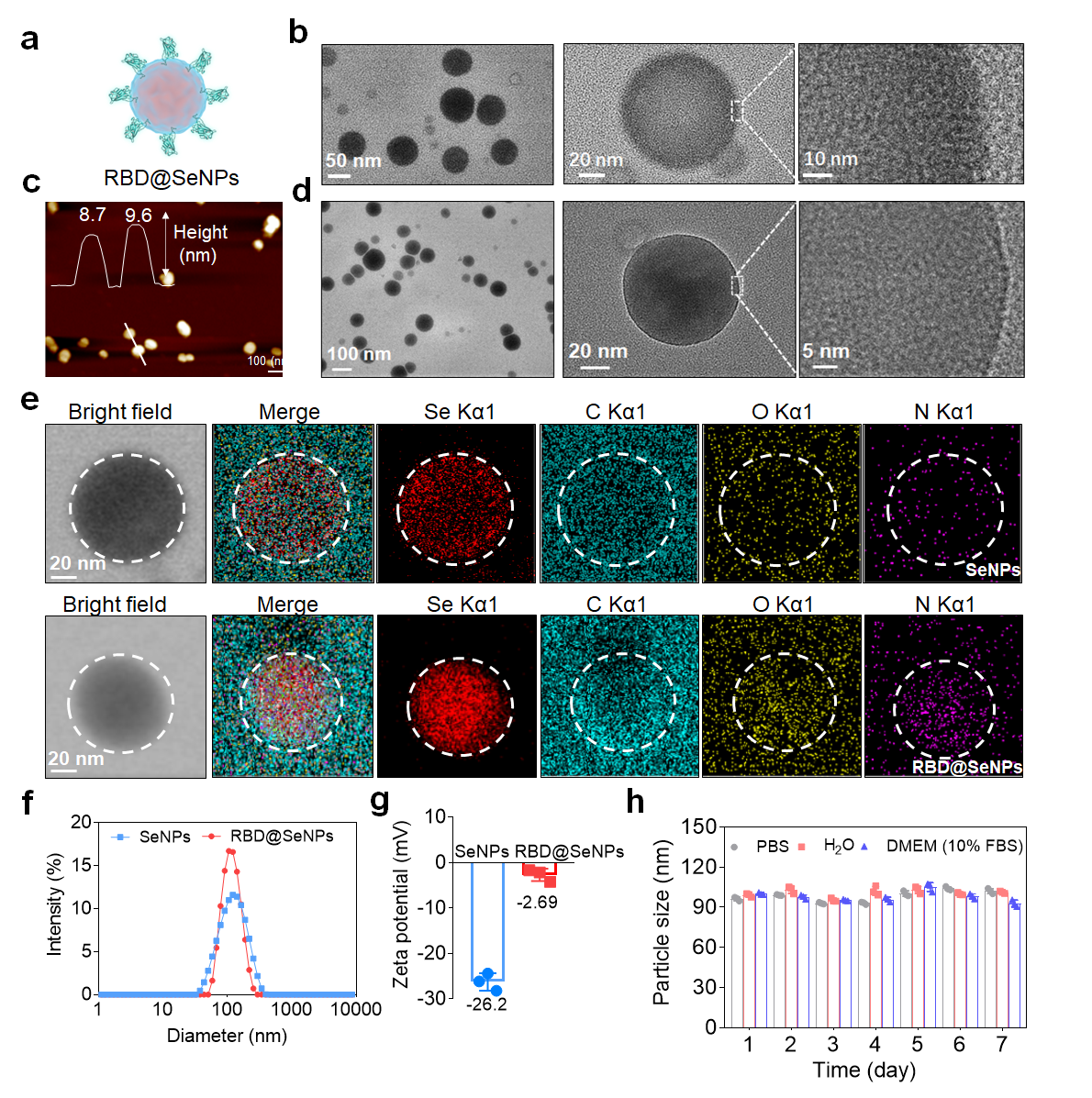


Fig. S3 Characterizations of Se nanoadjuvant and its complex with RBD. a Schematic illustration of Se nanoadjuvant complexed with RBD antigen. b TEM image of LET-SeNPs. AFM image and line scans (c), and TEM image (d) of RBD@SeNPs. e HRTEM images of LET-SeNPs and RBD@SeNPs. f Size distribution of SeNPs and RBD@SeNPs. g Zeta potentials of SeNPs and RBD@SeNPs. h Stability of RBD@SeNPs in PBS, H_2_O and DMEM (containing 10% FBS) for 7 days using DLS analysis. Data are expressed as the mean ± SD, n = 3 per group.

**Fig. S4 Effects of Se nanoadjuvant and the nanovaccine on the maturation of dendritic cells (DCs) and its regulation of toll-like receptors (TLRs) expressions.** The effects of Se nanoadjuvant, RBD and RBD@SeNPs on the maturation of bone marrow-derived dendritic cells (**a**) and cytokine (IL-12) production (**b**). The *in vivo* effects of Se-based vaccine RBD@SeNPs (**c**) and S1@SeNPs (**d**) on the maturation of DCs cells in inguinal lymph nodes. **e** The effects of Se nanoadjuvant on the mean fluorescence intensity of TLR1/2/4 on the surface of BMDCs using flow cytometry analysis. Data are expressed as the mean ± SD, n = 3 per group in **a** - **c** and **e**. n = 4 in **d**.


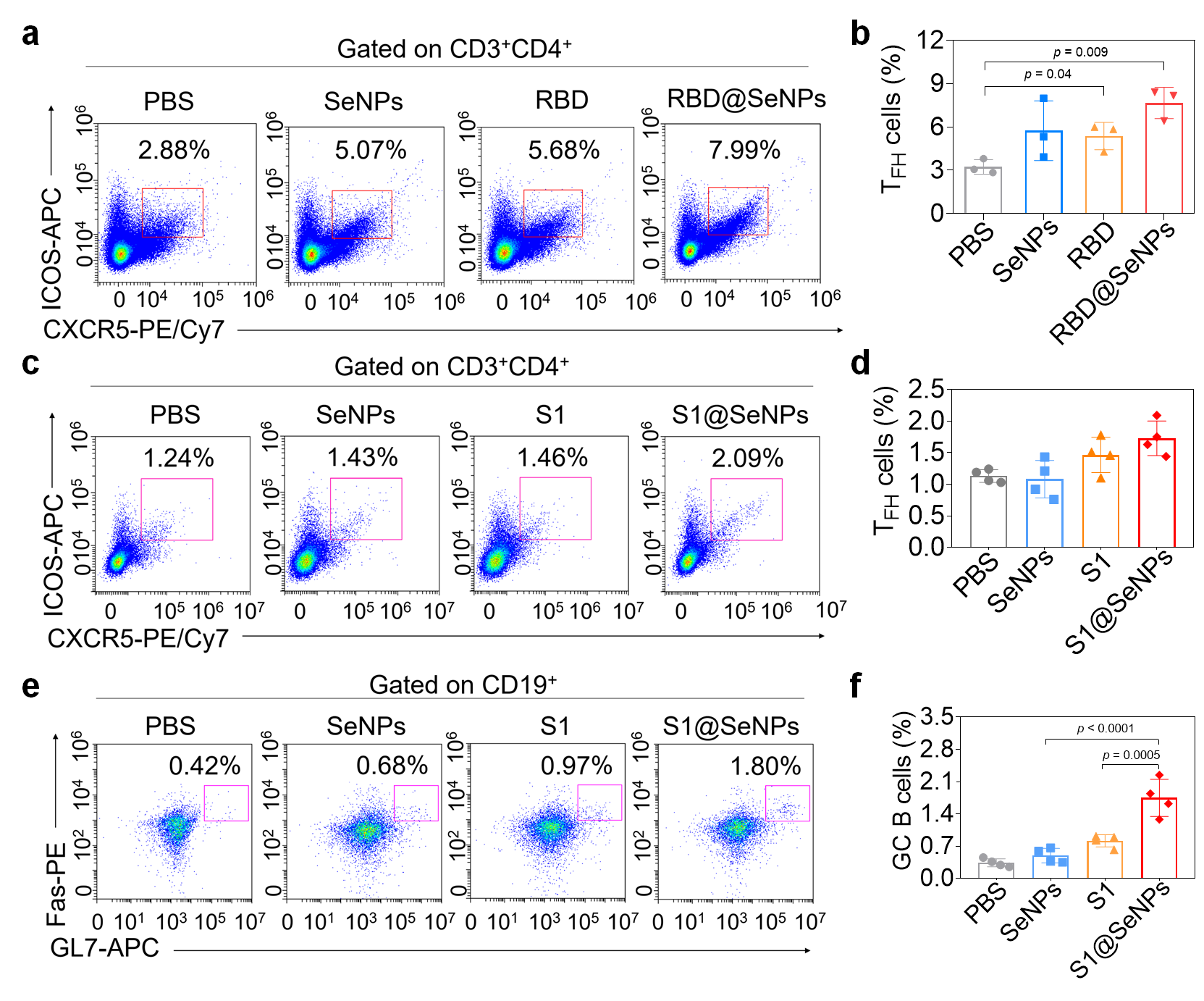
Fig. S5 Se nanoadjuvant could effectively enhance the immunogenicity of RBD or S1 spike protein antigen to induce more T_FH_ and GC B cells in splenocytes. The populations of T_FH_ cells (a - d) and GC B cells (e, f) in splenocytes of immunized mice at day 7 post vaccination. Data in (b) is expressed as the mean ± SD, n = 3 per group. Data in (d) and (f) are expressed as the mean ± SD, n = 4 per group.

 Fig. S6 Schematic diagram of RBD@SeNPs vaccination (a) and its effects on humoral immunity. Immunized mice were sacrificed at day 7 and 21 post final immunization, respectively. Then, the serum was collected for the titer and neutralization efficacy analysis of RBD-specific antibodies. The titer of IgM was determined by ELISA assay. Sera were collected at day 7 (b) and day 21 (c) post final immunization. d Th1/Th2 biased immune response analysis by determining the ratio of IgG2a to IgG1. Data are expressed as the mean ± SD, n = 4 per group.

Fig. S7 Representative fluorescence images of 293T ACE2 cells incubated with pseudovirus which pre-treated with serum collected at day 7 and day 21 post final immunization from different groups. Scale bar, 200 μm.

 Fig. S8 Quantitative analysis of SPS2, SEP15, GPX1, GPX3 and SELH mRNA expression level in splenocytes at day 7 post final immunization. Data are expressed as the mean ± SD, n = 3 per group.


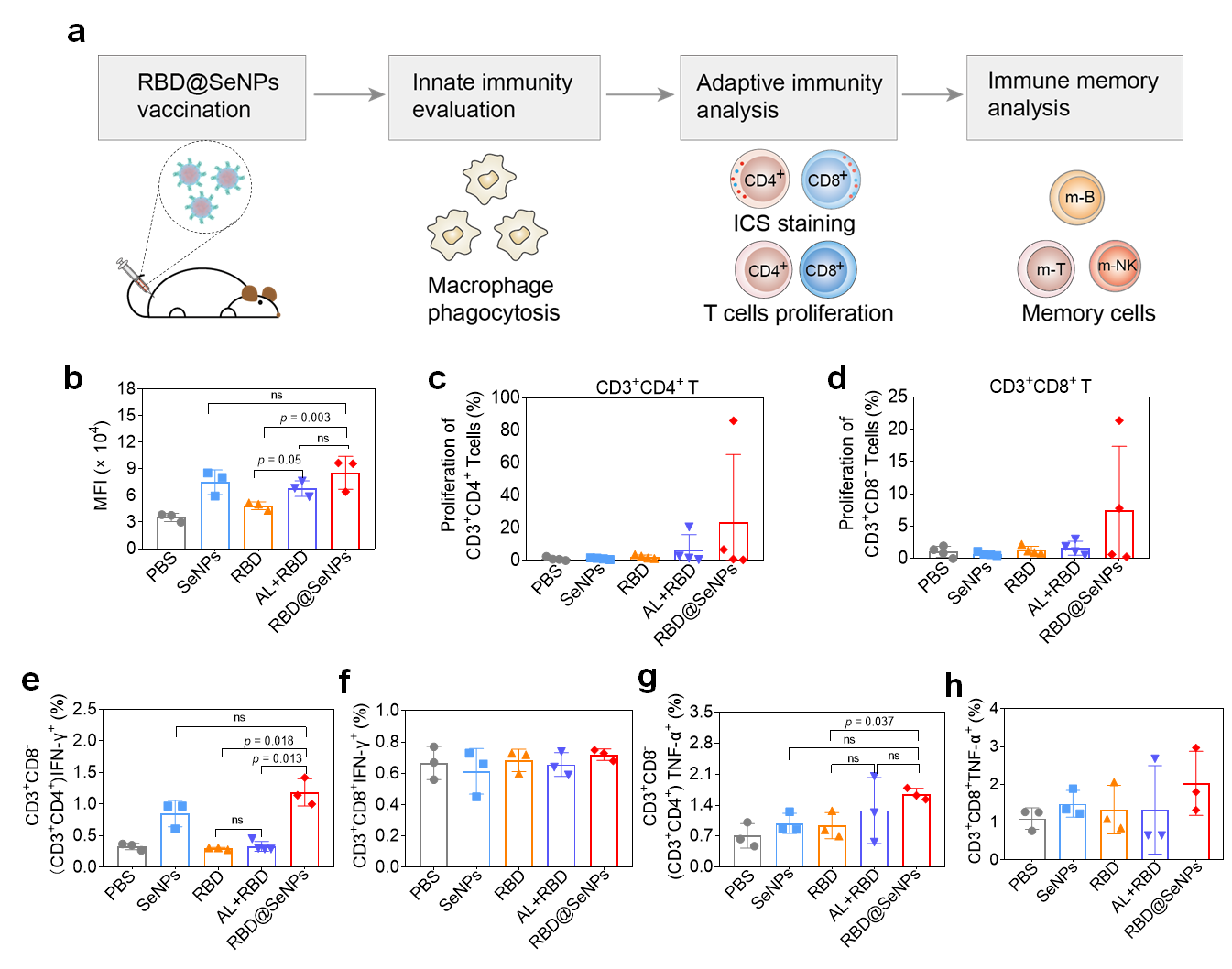


**Fig. S9 Se nanoadjuvant-based vaccine boosts innate immunity and Th1-biased cellular immunity. a** Schematic illustration of RBD@SeNPs vaccination and its effects on innate, adaptive immune responses and immunological memory. **b** The effects of different vaccinations on the phagocytosis capability of macrophages from immunized mice. Peritoneal macrophages were collected at day 7 after the final vaccination and incubated with fluorescence latex beads (100 nm, 2.5 μg/mL) overnight. The phagocytosis capabilities were determined by quantifying the intracellular fluorescence intensity using flow cytometry assay. Data are expressed as the mean ± SD, n = 3 per group. The effects of different vaccinations on the proliferation capabilities of CD3^+^CD4^+^ (**c**) and CD3^+^CD8^+^ T lymphocytes (**d**). Data are expressed as the mean ± SD, n = 4 per group. The effects of different vaccinations on the expression of IFN-γ (**e, f**), TNF-α (**g, h**) in CD3^+^CD8^-^ cells and CD3^+^CD8^+^ using intracellular staining assay. Splenocytes from immunized mice were collected at day 7 post final immunization and stimulated with 5 μg/mL recombinant RBD antigen for 6 h. Then, splenocytes were stained with specific antibodies against cytokines. Data are expressed as the mean ± SD, n = 3 per group.

 **Fig. S10** **The effects of Se nanoadjuvant and its complex with RBD antigen on immunological memory responses.** (**a-b**) The effects of different vaccinations on the populations of memory T lymphocytes. Central memory T cells in CD8^+^ T cells (**a**), CD4^+^ T cells (**c**) and effector memory T cells in CD8^+^ T cells (**b**), CD4^+^ T cells (**d**) were examined by staining with specific antibodies at day 21 after the last immunization. The effect of different vaccinations on the population of memory B cells in spleen (**e**) and lymph node (**f**). Data are expressed as mean ± SD, n = 4 per group.


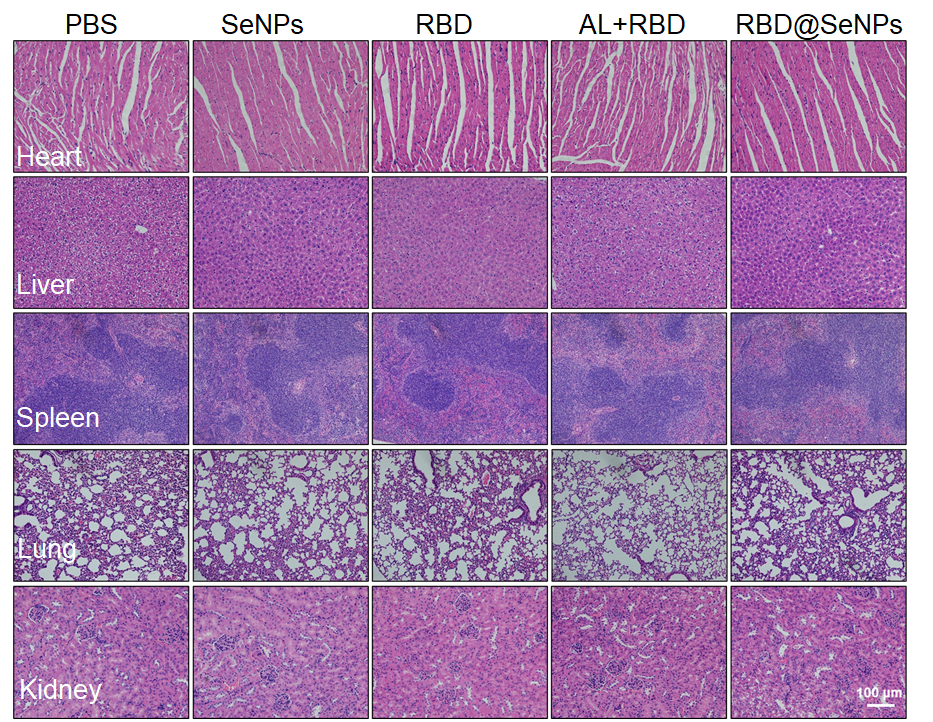


**Fig. S11 Histopathological analysis of the major organs in mice of different vaccinations.** After the last vaccination at day 21, mice were sacrificed and heart, liver, spleen, lung and kidney were collected and subjected for H&E analysis. Scale bar, 100 μm.

**Fig. S12** Expression of IL-4 and IL-21 in serum from mice at day 7 (**a, b**) and 21 (**c, d**) post final vaccination. Data are expressed as mean ± SD, n = 4 per group.


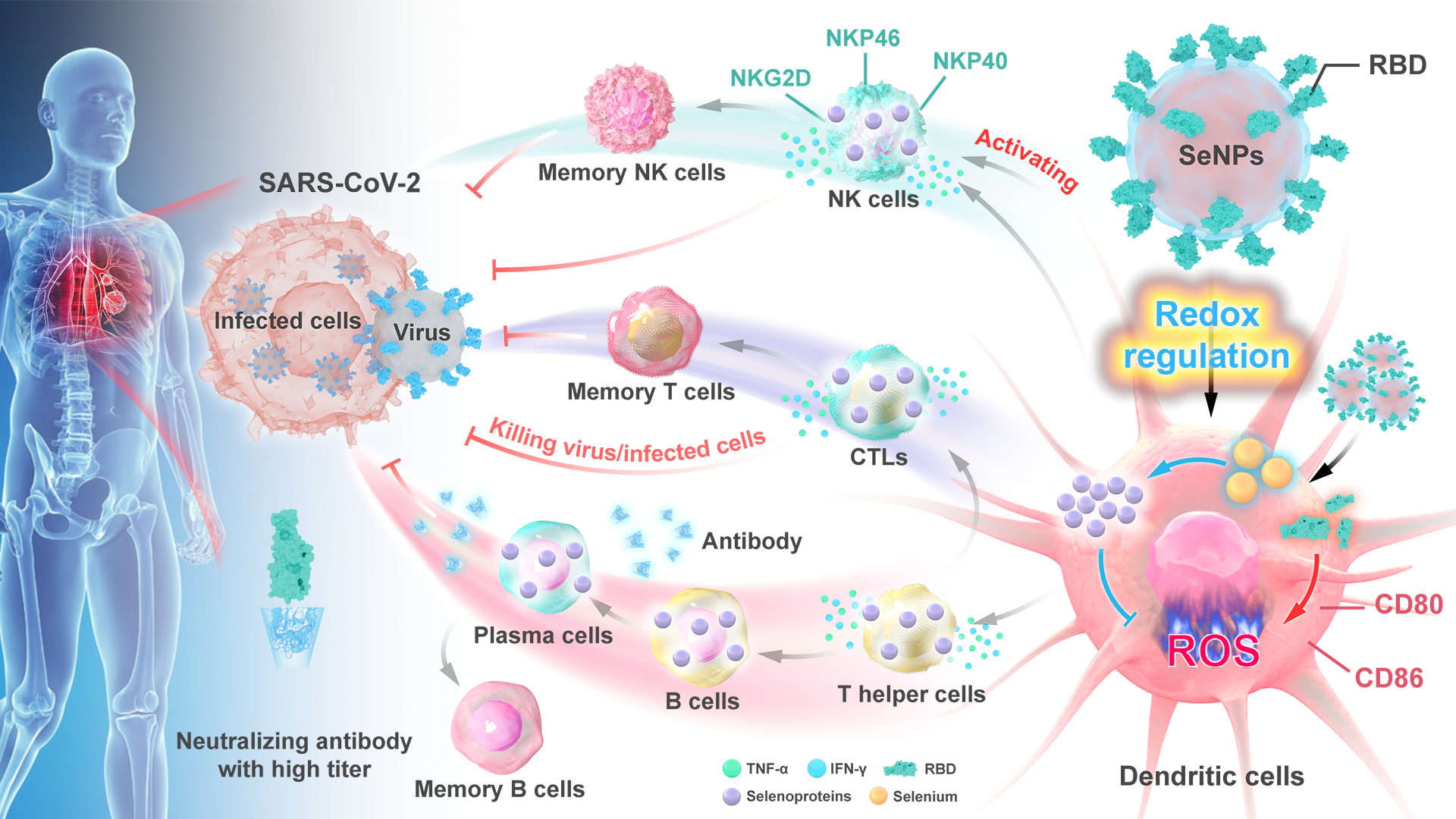
**Fig. S13** Schematic diagram of Se nanoadjuvant and its complex with RBD antigen in activating and regulating the redox balance within immune cells to elicit robust innate immunity, Th1-biased cellular immunity and neutralizing antibody with high titer for combating virus infection and its underlying mechanism.

Table S1. The primers sequence of selenoproteins.

| **Genes** | **5’-3’ sequence** | |
| --- | --- | --- |
| SELH | Sense | GGAAGAAAGCGTAAGGCGGG |
|  | Antisense | GGTTTGGACGGGTTCACTTGC |
| TRXR1 | Sense | CCTATGTCGCCTTGGAATGTGC |
|  | Antisense | ATGGTCTCCTCGCTGTTTGTGG |
| TRXR2 | Sense | GGCAACAGGGTGATGATCTTC |
|  | Antisense | CTGGAAAGTTCGGTCACATCC |
| TRXR3 | Sense | CTTTGCAAGATGCCAAGAAA |
|  | Antisense | TCATGGCCTCCCAGTTGT |
| GPX1 | Sense | ACAGTCCACCGTGTATGCCTTC |
|  | Antisense | CTCTTCATTCTTGCCATTCTCCTG |
| GPX2 | Sense | GCCTCAAGTATGTCCGACCTG |
|  | Antisense | GGAGAACGGGTCATCATAAGGG |
| GPX3 | Sense | ATTTGGCTTGGTCATTCTGG |
|  | Antisense | CCACCTGGTCGAACATACTTG |
| GPX4 | Sense | TCTGTGTAAATGGGGACGATGC |
|  | Antisense | TCTCTATCACCTGGGGCTCCTC |
| SEP15 | Sense | GCTGTCAGGAAGAAGCACAA |
|  | Antisense | TTTTCATCCGCAGACTTCAA |
| SPS2 | Sense | ACCGACTTCTTTTACCCCTTGG |
|  | Antisense | TCACCTTCTCTCGTTCCTTTTCAC |
| GAPDH | Sense | GGGAAGCCCATCACCATCT |
|  | Antisense | CGGCCTCACCCCATTTG |

**Original western blot bands for Fig 1f**
